# Predicting Post-Stroke Somatosensory Function from Resting-State Functional Connectivity: A Feasibility Study

**DOI:** 10.3390/brainsci11111388

**Published:** 2021-10-22

**Authors:** Xiaoyun Liang, Chia-Lin Koh, Chun-Hung Yeh, Peter Goodin, Gemma Lamp, Alan Connelly, Leeanne M. Carey

**Affiliations:** 1 Neurorehabilitation and Recovery, Florey Institute of Neuroscience and Mental Health, Melbourne, VIC 3084, Australia; clkoh@gs.ncku.edu.tw (C.-L.K.); peter.goodin@unimelb.edu.au (P.G.); g.lamp@latrobe.edu.au (G.L.); l.carey@latrobe.edu.au (L.M.C.); 2 Victorian Infant Brain Studies (VIBeS) Group, Murdoch Children’s Research Institute, Melbourne, VIC 3052, Australia; 3 Department of Occupational Therapy, Social Work and Social Policy, School of Allied Health Human Services and Sport, La Trobe University, Melbourne, VIC 3086, Australia; 4 Department of Occupational Therapy, College of Medicine, National Cheng Kung University, Tainan 701, Taiwan; 5 Imaging Division, Florey Institute of Neuroscience and Mental Health, Melbourne, VIC 3084, Australia; jimmy.chyeh@gmail.com (C.-H.Y.); alan.connelly@florey.edu.au (A.C.); 6 Institute for Radiological Research, Chang Gung University and Chang Gung Memorial Hospital, Taoyuan 33302, Taiwan; 7 Department of Psychiatry, Chang Gung Memorial Hospital, Linkou Medical Center, Taoyuan 33305, Taiwan; 8 Department of Psychology and Counselling, School of Psychology and Public Health, La Trobe University, Melbourne, VIC 3086, Australia

**Keywords:** functional connectivity, machine learning, regression, predictive modelling, stroke, somatosensory function

## Abstract

Accumulating evidence shows that brain functional deficits may be impacted by damage to remote brain regions. Recent advances in neuroimaging suggest that stroke impairment can be better predicted based on disruption to brain networks rather than from lesion locations or volumes only. Our aim was to explore the feasibility of predicting post-stroke somatosensory function from brain functional connectivity through the application of machine learning techniques. Somatosensory impairment was measured using the Tactile Discrimination Test. Functional connectivity was employed to model the global brain function. Behavioral measures and MRI were collected at the same timepoint. Two machine learning models (linear regression and support vector regression) were chosen to predict somatosensory impairment from disrupted networks. Along with two feature pools (i.e., low-order and high-order functional connectivity, or low-order functional connectivity only) engineered, four predictive models were built and evaluated in the present study. Forty-three chronic stroke survivors participated this study. Results showed that the regression model employing both low-order and high-order functional connectivity can predict outcomes based on correlation coefficient of *r* = 0.54 (*p* = 0.0002). A machine learning predictive approach, involving high- and low-order modelling, is feasible for the prediction of residual somatosensory function in stroke patients using functional brain networks.

## 1. Introduction

Stroke is now the second largest cause of death and disability, with a lifetime risk of 1 in 4 [1]. There are more than 80 million people living with the consequences of stroke worldwide [1]. A good recovery of brain function, and motor and somatosensory function in particular, is crucial for regaining independence and quality of life for most people who have experienced a stroke [2,3,4]. However, there has been rare success in predicting patient’s recovery and outcome using clinical assessment alone [4,5].

Intuitively, neurological impairment following stroke is caused by damage to brain regions. Following this perspective, previous studies have mainly focused on the mapping of symptoms to a focal lesion. However, accumulating evidence has demonstrated that brain functional deficits can extend to remote connected brain areas [6,7]. This is consistent with recent evidence from us that structural connectivity remote from lesions correlates with somatosensory outcome post-stroke [8].

Neuroimaging biomarkers have clinical significance for research translation as they can provide clinically useful information when planning the personalized rehabilitation of a patient. While the benefits of employing neuroimaging predictors of outcome and recovery have been highlighted in a recent consensus statement, the relative lack of diagnostic and predictive biomarkers for somatosensory outcomes was also identified [6]. Functional biomarkers, including task-related activation and resting-state functional connectivity were considered as a developmental priority [6]. Predictive modelling of the association between somatosensory outcome and functional brain networks is indicated.

Brain connectomics is a field studying the topological characteristics of the brain network or ‘connectome’—the comprehensive map of the neural elements (nodes) and their inter-connections (edges) that constitute the brain. At a macroscale, two types of connectomes are commonly used, i.e., the functional and structural connectome using functional MRI (fMRI) and diffusion-weighted MRI (dMRI), respectively [9]. Increasing evidence supports that the brain should be modelled as an ensemble of functional networks rather than focusing on local functional brain areas. Human imaging data show strong associations between connectivity and outcome after stroke [8,10,11,12,13].

Resting-state functional connectivity has been emerging as a powerful tool to map the functional networks across the whole brain using either Blood-oxygen-level dependent (BOLD) fMRI [14] or arterial spin labelling perfusion fMRI [15,16]. Resting-state functional connectivity provides a direct and simple measure of regional interaction without any explicit task requirements, as is needed for task-based fMRI. While resting-state functional connectivity measures are mostly used at the group level, they could also provide personalized information at the individual level, which provides the potential of using single-subject data for individual diagnosis and prognosis. Distributed brain networks are shown to be involved in processing somatosensory information, including both hemispheres, primary and secondary somatosensory regions, and subcortical areas [17]. The extent to which an individual may experience interruption to one or more regions within this distributed brain network after stroke likely impacts the nature and severity of somatosensory deficits.

Certain brain subnetworks have been demonstrated to be compromised in stroke survivors following cortical or subcortical lesions, with dysfunction manifested in behavioral impairment [18]. For example, in our previous studies using task-related brain activation in stroke survivors with impaired touch sensation, evaluated using the Tactile Discrimination Test (TDT) [19], we showed distinct differences in patterns of brain activation between stroke survivors with varying lesion location [20] and in age-matched healthy controls [21] whilst performing a touch discrimination task in the scanner. We have also applied univariate analyses to explore the relationship between performance on the TDT and functional brain networks, using both resting-state and seed-based functional connectivity [13,22].

However, to date we have not been able to predict residual somatosensory function from interruption to brain networks, partly because of the complex interactions between brain regions and the limitations in the current methods interrogating these effects. Methodologically, these limitations could be attributed to the following reasons:

Firstly, conventional resting-state functional connectivity is the most popular technique for measuring functional connectivity; this is also referred to as low-order functional connectivity (LOFC) because it simply measures the temporal correlation of the resting-state fMRI time courses between any pair of brain regions. More recently, the concept of ‘high-order’ functional connectivity (HOFC) has been proposed to measure the “correlation of the correlation” [23,24]. In LOFC, each row (column) of the connectome encodes the correlation of BOLD time series between one area to all the other areas in the brain in a pairwise manner; in HOFC, each row (column) encodes the correlation between one row (column) to every other row (column) of the LOFC. In this way HOFC is designed to capture the important interactions among all related brain regions (e.g., the correlations among different edges in a network), essentially characterizing the high-order relationships between brain regions and networks by including global anatomical information. As demonstrated in Zhang and colleagues’ work [23,24], HOFC has been shown to be useful in providing ‘high-level’ information for brain disease studies and for building predictive models. Simultaneous estimation of both LOFC and HOFC might also be of value [25].

Second, whole-brain connectomics (region-based or voxel-wise) involves high-dimensional data, which essentially requires multivariate analysis. However, univariate analysis has been predominantly employed for such investigations in previous studies [13,26]. Therefore, it is expected that new insights into stroke recovery can be obtained by employing machine learning techniques due to their multivariate capabilities.

In the current study, we aim to investigate whether the residual somatosensory function of stroke survivors, estimated by TDT scores, can be predicted from resting-state functional connectivity using multivariate predictive modelling techniques. Given the observation that brain functional and structural disruptions can extend to remote connected brain areas [6,7,8], we hypothesize an association between post-stroke residual somatosensory function and functional brain connectivity based on predictive modelling using machine learning techniques, and that the strength of the relationship will be enhanced when high-order relationships are included. Specifically, we seek to identify the functional connectivity pathways that are closely associated with somatosensory impairment. This preliminary work might provide new insights to disruption of functional brain networks associated with impaired somatosensory function after stroke, thereby advancing the foundation for the development of biomarkers of somatosensory recovery and novel therapeutic interventions in the future.

## 2. Materials and Methods

### 2.1. Participants

Participants were recruited for the Connecting New Networks for Everyday Contact through Touch (CoNNECT) study (https://anzctr.org.au/Trial/Registration/TrialReview.aspx?id=364147, accessed date: 20 October 2021). Inclusion criteria included: (1) at least three months post first episode of stroke (ischaemic or haemorrhagic); (2) experiencing somatosensory impairment in the upper limb; (3) medically stable; (4) able to give informed consent; (5) able to comprehend simple instructions; (6) right-hand dominant. Exclusion criteria included: (1) a brainstem infarct; (2) previous neurological dysfunction; (3) history of impaired hand function; (4) peripheral neuropathy in upper limbs; (5) evidence of neglect on standard neuropsychological tests; (6) not suitable for MRI scanning. Informed consent was obtained from all participants and all protocols were approved by hospital and university Institutional Review Boards.

A total of 43 participants with chronic stroke were recruited, of which 2 were excluded due to excessive head motion and 1 removed due to significant signal dropout in the anterior region of the brain caused by an implant. As a result, 40 stroke survivors were included in the present study. Demographic and clinical details of participants are provided in Table 1.

### 2.2. Tactile Discrimination

The TDT test was performed within 48 h of the MRI scan and 2-weeks earlier. The average scores of the two baseline measures were used [19]. Participants are required to discriminate differences in finely graded plastic texture surfaces using the method of constant stimuli and a three-alternative forced-choice design. Five texture differences are each sampled across five test runs (i.e., twenty-five trials of triplet-textures in total). The area under the curve (AUC) score is then calculated to determine texture discrimination, after accounting for chance responses. The TDT has age-appropriate normative standards, high test-retest reliability, and good discriminative properties [19].

### 2.3. MRI Data Acquisition

All of the MRI data were collected on a 3T Siemens Tim Trio (Siemens, Erlangen, Germany) with a 12-channel head coil. Resting-state BOLD fMRI data were acquired with a gradient-echo echo-planar imaging (EPI) sequence using the following parameters: TR/TE= 3000/30 ms, voxel size = 3 mm isotropic, number of slices = 44, matrix size = 72 × 72, number of time points = 140. Participants were instructed to keep eyes closed and stay awake during the scan, which was further confirmed when scans were completed.

Anatomical images were acquired with a three-dimensional Magnetization-Prepared-Rapid-Gradient-Echo (MPRAGE) sequence, using the following parameters: TR/TE/TI = 1900/2.55/900 ms, flip-angle = 9°, 1 mm isotropic resolution, field-of-view = 256 × 256 mm^2^, 160 partitions. Two-dimensional T2-weighted fluid attenuation inverse recovery sequence (T2 FLAIR) images were acquired axially for delineation of infarcts, with the following parameters: TR/TE = 6000/388 ms, voxel size = 0.5 × 0.5 × 3 mm^3^.

### 2.4. Lesion Mask Creation

Lesion Masks were manually drawn on 2D Axial FLAIR images using MRIcron (https://www.nitrc.org/projects/clinicaltbx/, accessed date: date: 3 October 2021) by a trained neuroimaging researcher. These lesion masks were quality-checked and modified as necessary by an experienced neurologist to ensure accurate delineation of the infarct.

### 2.5. Data Analysis

All of the MR image pre-processing was conducted using the SPM8 toolbox (https://www.fil.ion.ucl.ac.uk/spm/software/spm8/, accessed date: 2 September 2020) with the following pre-processing steps: (1) realigning the BOLD data; (2) co-registering the BOLD data obtained in step (1) to T2 FLAIR space; (3) co-registering the lesion mask to T1 anatomical space; (4) applying segmentation-normalization to T1 and lesion mask obtained in step (3) by using the clinical toolbox for SPM (https://www.nitrc.org/projects/clinicaltbx/, accessed date: 3 October 2021); (4) co-registering the BOLD data obtained in step (2) to T1 space; (5) normalizing the BOLD data obtained in step (4) to MNI template; (6) smoothing the BOLD data obtained in step (5) with smoothing kernel of FWHM = [6 6 6]. Independent component analysis was then applied to pre-processed fMRI data using FSL’s Melodic tool (https://fsl.fmrib.ox.ac.uk/fsl, accessed date: 5 August 2021). Artifact-related independent components were manually identified and removed by applying FSL’s fsl-regfilt command.

### 2.6. Construction of Functional Connectomes

Initially, the automated anatomical labelling (AAL) template was employed to parcellate the entire brain into 116 regions [27]. Subsequently, the cerebellum was excluded due to the potential issue of low fidelity of signal measurement with BOLD signal from cerebellum [28], thus yielding the 90-region AAL parcellation. Region-wise time courses were calculated by averaging voxel-wise time courses across all brain regions, followed by the computation of connection strength by using Pearson correlation, yielding a connectivity matrix of size 90 × 90 for each subject. Firstly, LOFCs were constructed to measure the relationship between brain region pairs using Pearson correlation between any pair of regions. In addition, HOFCs [23] were also computed to capture second-level relationships using inter-regional similarity of the FC topographical profiles, i.e., measuring the ‘correlation of the correlation’ described previously.

### 2.7. Regression Predictive Modelling

**Figure 1:**
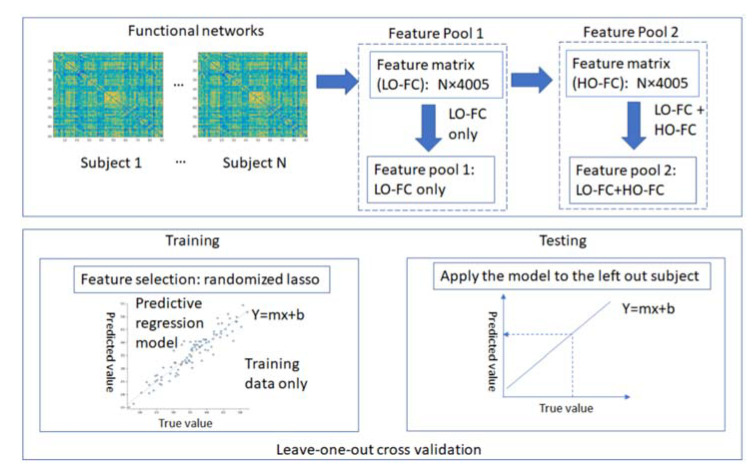
Flow chart of the framework.

To predict residual somatosensory function (using the TDT scores as the indicator) from participants who had experienced a stroke, functional connectomes were employed as potential features. Specifically, the predictive modelling was conducted with two regression models: linear regression (LR), and support vector regression (SVR) with linear kernels. The prediction framework is described as follows (see Figure 1 for flow chart of the framework).

#### 2.7.1. Feature Engineering

Feature engineering aims to transform data into features that can optimally train the predictive models, resulting in improved model accuracy on unseen data. Predictive outcomes could be largely affected by the obtained features. Importantly, the extraction of more accurate features leads to higher flexibility in choosing models, or even simplifying model selection. In this study, feature engineering employed the following two feature pools: (1) LOFC; and (2) the combination of LOFC and HOFC (denoted as LOFC + HOFC hereafter).

As for neuroimaging data, they often have a much larger dimension size than the sample size, which are easily subject to overfitting. To address this issue, sparse feature selection approaches are of particular interest, such as the Least Absolute Shrinkage and Selection Operator (LASSO) approach [29]. Recently, a stability selection approach was proposed to solve the notoriously difficult problem of variable selection. For high-dimensional data, stability selection was demonstrated to be very useful in selecting sparse variables. The robustness of stability selection has been already demonstrated in estimating sparse network edges from high-dimensional brain connectome data [30,31]. Therefore, the randomized LASSO with stability selection was conducted to select optimal and robust features for predictive modelling using sklearn (scikit-learn.org, accessed date: 20 October 2021).

#### 2.7.2. Model Validation: Leave-One-Out Cross-Validation

Given that a limited number of stroke participants (*N* = 40) were available for training predictive models, a nested leave-one-out cross-validation approach was employed, with inner- and outer-loop iterations. In each outer-loop iteration, one participant is retained as the test set, and the remaining N-1 participants were considered as the training set; feature selection was applied only to the training set ensuring blind test condition. The inner-loop leave-one-out cross-validation included N-1 subjects inputted from the outer-loop iterations, with N-2 subjects as the training set and one participant retained as the test set, with which hyperparameters tuning for support vector regression (C and gamma) was conducted in the inner-loop iteration. Optimal hyperparameters were not fully consistent across the N repetitions due to relatively low signal to noise ratio of fMRI data. As such, a majority vote was employed to choose the final optimal hyperparameters, i.e., a hyperparameter was designated as the final optimal one if it was estimated as the optimal hyperparameter (i.e., achieving the highest correlation coefficient (*r*) between predicted and true TDT scores) by the most folds. The trained predictive model on each training set was then applied to the retained participant, predicting the TDT score of that participant.

With the nested leave-one-out cross-validation conducted, the TDT score of each participant was predicted using a specific model and obtained features associated with the training set. The predicted TDT scores were then correlated with true TDT scores, with a higher correlation coefficient indicating better performance in predicting TDT scores. Finally, the prediction outcomes were calculated and compared between the two regression models, linear regression, and support vector regression, using either LOFC or LOFC + HOFC as the potential feature pools.

#### 2.7.3. Final Model Building

Ideally, selected features are expected to be consistent across all folds while conducting leave-one-out cross-validation. However, selected features are not necessarily the same among all folds, in which case a final optimal feature set is needed for constructing the final model. To achieve this goal, we empirically selected only those that were selected as features from not less than half of the folds from the leave-one-out cross-validation implementation. The selected set of features were subject to further feature selection on all stroke participants using randomized LASSO as described above. The final model was trained on all available participants, producing the final model for future prediction.

## 3. Results

Our regression prediction modelling results have shown that the accuracy of predicting residual sensory function from resting-state functional connectivity using 90-region AAL parcellation is significantly better than by chance, i.e., *p* < 0.05 for both regression models (i.e., LR and SVR) with either LOFC or LOFC + HOFC as feature pools, as shown in Table 2.

**Figure 2:**
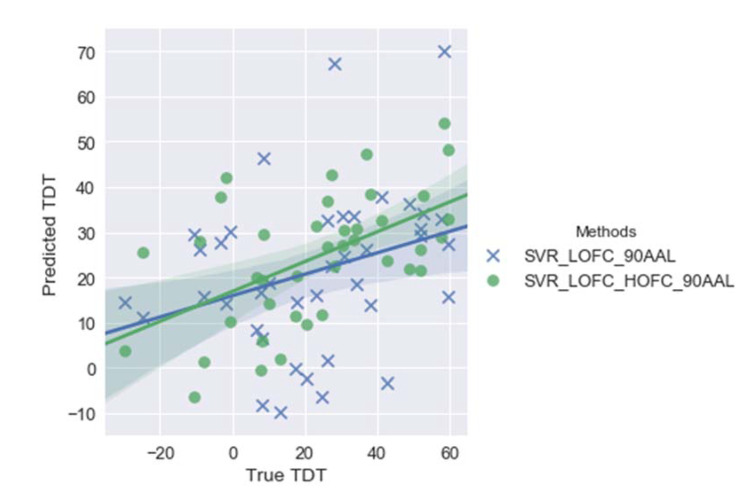
Plots of predicted vs. true Tactile Discrimination Test (TDT) scores by employing support vector regression (SVR) with either LOFC or LOFC + HOFC with feature pools (cerebellum excluded). Correlation coefficients, *r*, between predicted and true TDT scores are 0.54 and 0.31 for SVR_LOFC_HOFC_90AAL, SVR_LOFC_90AAL, respectively. Note: SVR_LOFC_HOFC_90AAL represents the SVR model using both LOFC and HOFC features based on automated anatomical labelling (AAL) atlas, while SVR_LOFC_90AAL represents the SVR model using LOFC features only based on AAL atlas. LOFC = low-order functional connectivity. HOFC = high-order functional connectivity.

When employing support vector regression as the regression method, the comparisons between 2 feature pools were conducted, i.e., LOFC vs. LOFC + HOFC. Figure 2 shows that while both predictive models (support vector regression with LOFC or support vector regression with LOFC + HOFC) can predict TDT scores with relatively high accuracy (i.e., with relatively high correlation coefficient values: 0.54 and 0.31, respectively), the support vector regression model with LOFC + HOFC outperforms support vector regression with LOFC only.

**Figure 3:**
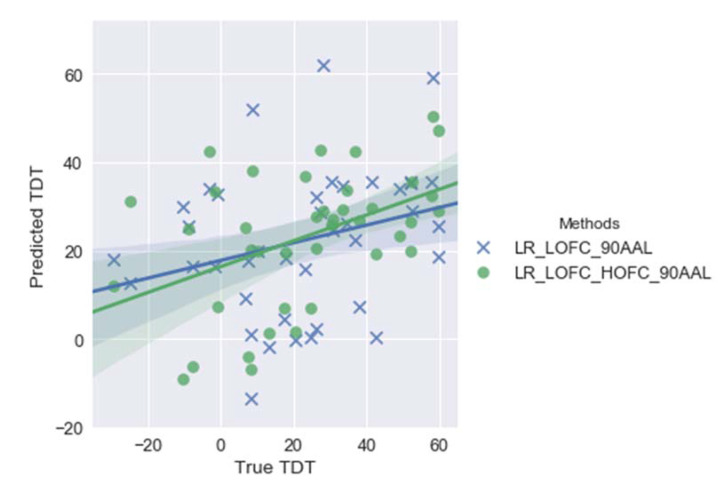
Plots of predicted vs. true Tactile Discrimination Test (TDT) scores by employing linear regression (LR) with either LOFC or LOFC + HOFC with feature pools (cerebellum excluded). Correlation coefficients, *r*, between predicted and true TDT scores are 0.45 and 0.28 for LR_LOFC_HOFC_90AAL and LR_LOFC_90AAL, respectively. Note: LR_LOFC_HOFC_90AAL represents the LR model using both LOFC and HOFC features based on automated anatomical labelling (AAL) atlas, while LR_LOFC_90AAL represents the LR model using LOFC features only based on AAL atlas. LOFC = low-order functional connectivity. HOFC = high-order functional connectivity.

**Figure 4:**
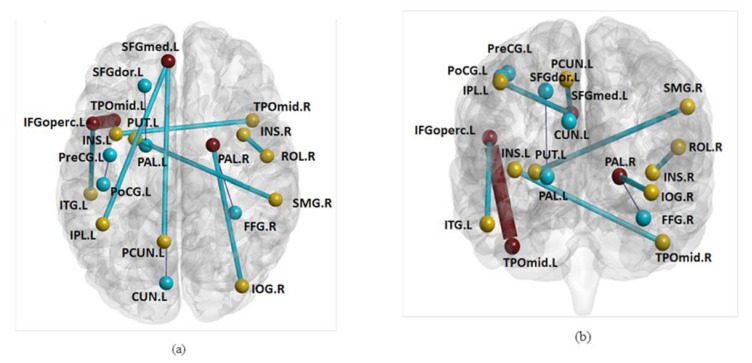
Selected features (i.e., functional network edges) that have been employed to build final models. Selected features are mapped onto automated anatomical labelling (AAL) atlas in: (**a**) axial view and (**b**) coronal view. Note: Blue and yellow nodes are involved in LOFC and HOFC, respectively, whereas the brown node (e.g., SFGmed.L) is involved in both LOFC and HOFC; network edges identified as features are classified into 3 categories: (1) An edge is identified as a feature from LOFC only (black thin edges); (2) An edge is identified as a feature from HOFC only (blue edges); (3) An edge is identified as a feature from both LOFC and HOFC (brown thick edges). LOFC = low-order functional connectivity. HOFC = high-order functional connectivity. Names and corresponding abbreviations of the brain regions are presented in the Appendix A.

With respect to linear regression, similar trends to the support vector regression have been identified. Specifically, the employment of both LOFC and HOFC provides higher accuracy in predicting TDT scores (Figure 4). Nevertheless, compared with support vector regression, the regression models with linear regression achieve a lower overall accuracy, i.e., lower *r* values and higher *p* values (see Figure 2 and Figure 3 and Table 2).

Based on the model comparisons, the model achieving highest accuracy, i.e., the support vector regression model with LOFC + HOFC achieving highest r value, was selected as the best model, with which the optimal features were also identified. Specifically, 13 functional network edges were identified as important features that can predict TDT scores (Figure 4). The brain regions connected by these edges include: Left precentral gyrus; left superior frontal gyrus-dorsal part; left inferior frontal gyrus-opercular; right rolandic operculum; left superior frontal gyrus-medial part; left insula; right insula; left cuneus; right inferior occipital gyrus; right fusiform gyrus; left postcentral gyrus; left inferior parietal lobe; right supramarginal gyrus; left precuneus; left putamen; left pallidum; right pallidum; left temporal pole-middle; right temporal pole-middle; left inferior temporal gyrus.

## 4. Discussion

In this preliminary study, we investigated the feasibility of applying a machine learning approach to predict somatosensory impairment after stroke using resting-state functional connectivity data. Specifically, texture discrimination of the hand was measured using the TDT [19]. As the brain is organized into a set of distributed networks, focal stroke lesions could affect functions in remote but connected regions [6,7,8], which suggests that stroke impairment could be better understood by using a brain network model. As such, stroke impairment may be modelled relative to the disruption of network edges (i.e., connection strength), in which important features (functional network edges) can be identified with a proposed machine learning approach. Our results demonstrate the potential of the proposed regression predictive approach for predicting residual somatosensory function, i.e., tactile discrimination function of the hand, from participants with stroke using a brain networks approach. These relationships are important to be established as a foundation for biomarkers at a point in time (for diagnosis/classification), at future time (for prediction), or in association with evidence of neuroplastic changes associated with spontaneous and/or treatment-facilitated recovery (biomarker of mechanisms underlying recovery). Further, linking the underlying impairment with disruption to functional brain networks provides new insights that can be used to inform the development of neuroscience-based interventions. For example, knowledge that the behavioral TDT outcome is better predicted with inclusion of high-order relationships in the model suggests the potential explanatory value of interactions among not only brain regions, but also between brain networks. It also identifies the specific regions and networks involved, highlighting the value of global anatomical information rather than relying on focal lesion alone.

The main difficulty of utilizing brain networks in the predictive modelling of stroke outcomes lies in the fact that there are a greater number of features (*p*~thousands of features) than the number of samples (*n* ~ tens to hundreds of subjects), i.e., *p >> n*, which usually leads to overfitting issues. In addition, the reliability of potential features could largely affect the performance of the predictive models. Yet, these are real and common issues in clinical studies. Nevertheless, the combination of LOFC with HOFC achieved higher performance of predictive modelling (*r* = 0.54 and 0.45) than that with LOFC only (*r* = 0.31 and 0.28). Our findings suggest that feature engineering, i.e., the process of generating features that can be employed to build predictive models, is a crucial step for successfully predicting stroke outcomes using the resting-state functional connectivity data.

Given the common issue of *p >> n* for neuroimaging data, we attempted to reduce the number of features, i.e., *p*, so that the overfitting issue could be alleviated. Firstly, to achieve this goal, feature selection was explicitly implemented so that only a limited number of the most important features were selected. Our results showed that the employment of the stability selection approach can effectively extract a small number of important features, i.e., sparse networks were obtained, therefore alleviating the overfitting issue. Secondly, with the consideration of the relatively small number of participants, we elected to use simple models rather than complex models (random forest etc.), i.e., only linear regression (LR) and support vector regression (SVR) models were employed. Results have demonstrated the outperformance of support vector regression with linear kernel (i.e., r = 0.54 and 0.31 for LOFC + HOFC vs. LOFC only) over linear regression (i.e., r = 0.45 and 0.28 for LOFC + HOFC vs. LOFC only). Such observed differences between support vector regression and linear regression might be well explained by the “winner-take-all” property of linear regression, i.e., when two features are highly correlated, the weight assigned to the second feature is close to 0, whereas the weights of both features should be similar. Therefore, support vector regression is preferred over linear regression for brain functional connectivity studies, i.e., functional connections highly correlated should have similar weights.

As a final optimal model for predicting residual somatosensory function (TDT scores), those 13 features identified are considered crucial in somatosensory information transfer processing. Such connectivity roles are well supported by the comprehensive functions of involved brain regions. Interestingly, those regions can be roughly classified into three categories according to their respective roles in the literature: (i) brain regions involving basic somatosensory and/or motor functions (low level sensory perception): precentral gyrus and postcentral gyrus [32]; (ii) brain regions performing multisensory information integration: inferior parietal lobule [33], insula [34], and precuneus [35]; (iii) brain regions responsible for multisensory information processing (high-order cognitive processing): superior frontal gyrus—dorsal and medial parts [36], rolandic operculum [37], cuneus [38], inferior occipital gyrus [39], fusiform gyrus [38], supramarginal gyrus [40], putamen [41], pallidum [42], temporal pole—middle [43], and inferior temporal gyrus [44]. Specifically, those brain regions in multisensory information integration (category ii) act as bridges and transfer information from regions involved in basic somatosensory and/or motor functions (category i) to regions in multisensory information processing (category iii).

To the best of our knowledge, this is the first attempt to employ multivariate modelling techniques as a tool to reveal the relationship between somatosensory impairment after stroke and brain networks. Compared with previous studies that commonly used univariate analysis [13], our study employed a multivariate approach to identify the relationship between whole-brain connectome and stroke impairment in somatosensory function. Further, we adopted both low-order and high-order FC as a feature pool. This approach achieved higher correlation scores relative to the model that only employed low-order FC as a feature pool, which is consistent with the findings of previous studies [23,24]. Furthermore, this multivariate approach provides a potential approach to identify important network edges from the whole-brain network that significantly affect the residual somatosensory function. Knowledge that the behavioral TDT outcome is better predicted with inclusion of high-order relationships in the model suggests the potential explanatory value of including an index of anatomical edges. Further, evidence of the feasibility of establishing a relationship between brain regions and brain networks and touch discrimination function at a single point in time can provide a foundation for future definition of neuroimaging biomarkers for predicting stroke recovery and rehabilitation if very large data sample size is available.

### Limitations and Future Work

There are a few methodological limitations that need to be considered. Firstly, the limitation of the current study is that only a limited number of participants were recruited and analyzed, i.e., 40 in our final analysis, whereas machine learning typically requires a large amount of data. This could lead to the following issues: (1) the effect of overfitting is likely strong; (2) outliers might have much more adverse effect on prediction; (3) noise becomes more of an issue. However, clinical neuroimaging data often have limited number of participants, often in the magnitude of several tens [45]. In the future, multicenter studies might be leveraged so that the potential of machine learning techniques can be largely realized.

Secondly, the focus of the current study was a cohort of stroke survivors, in which lesions are distributed across the brain. It remains an open question as to how to deal with those lesioned brain regions. In this study, lesion masks were applied when normalizing the brain using the Clinical Toolbox in SPM (https://github.com/neurolabusc/Clinical), which accounted for the lesions during the preprocessing. We did not apply a lesion mask when constructing connectomes. This is because we preferred not to exclude the potential disrupted connections, which would otherwise be removed completely by applying a lesion mask. We reasoned that while structural damage is likely to impact functional connectivity, the relationship between structure and function is not necessarily 1:1, and functional connectivity may spread over multiple anatomical paths [46]. To our best knowledge, there is no consensus on how to address this issue optimally. Future studies may be required to investigate the optimal way of dealing with the lesions.

While our results show that the accuracy of predicting TDT scores from brain connectomes using regression analysis is significantly better than by chance, the best predictive model with selected features can explain only ~30% variance, which likely indicates that there might still be large room for improvement. Practically, given that only functional connectivity has been used as a tool to investigate the relationship among brain networks and stroke impairment, structural connectivity (or connectomes from other imaging modalities) could be included to further improve the predictive outcome, especially as it is complementary to functional connectivity. Alternatively, as proposed in a previous study [47], functional connectivity and structural connectivity could be combined into a single image, which could provide predictive modelling with features that contain complementary information from both modalities. Our work in the future will focus on how to improve the prediction outcomes via multi-modal connectomes.

## 5. Conclusions

This preliminary study provides a multivariate approach for investigating the relationship between stroke impairment and functional brain networks. Our findings demonstrate the feasibility of predicting post-stroke residual somatosensory function using resting-state functional connectivity and predictive modelling. Specifically, by employing a robust feature selection approach, i.e., randomized LASSO based on stability selection, a small number of the most important features (brain network edges) are selected. Using this approach, stroke impairment may be more directly related to brain networks when employing machine learning techniques. The role of interconnected brain regions involved with basic somatosensory and/or motor functions, multisensory information integration, and multisensory information processing were revealed in association with clinical somatosensory impairment. While our study focuses on somatosensory impairment, this approach could be generalized to other stroke impairments as well. Thus, this study develops a possible avenue for linking stroke impairment to functional brain networks. However, the validity of the proposed approach needs to be evaluated with so-called ‘big data’, which is likely to facilitate the extraction of neuroimaging biomarkers, providing clinically useful information when planning the personalized rehabilitation of a patient.

## Figures and Tables

**Table 1:** Demographics of the participants.

	Stroke (*n* = 40)
Sex, F/M	11/29
Mean age, years (SD)	51.8 (13.2)
Stroke type, I/H	32/8
Stroke chronicity, mean months (SD)	18.6 (22.1)
Side of lesion, L/R	20/20
Lesion location, C/S/M	19/12/9
Lesion size (c.c.) [Q1, Q3]	[12.5, 70.1]
TDT contralesional affected hand, mean (SD) *	22.6 (23.2)
TDT ipsilesional hand, mean (SD)	65.9 (18.6)

I = ischemia; H = Haemorrhage; C = cortical lesion; S = subcortical lesion; M = mixed cortical and subcortical lesion; TDT = the Tactile Discrimination Test [19]; n = number of subjects; SD = standard deviation; Q1 = lower quartile; Q3 = upper quartile. * Criterion of abnormality for the TDT is 60.25 Area Under the Curve (AUC). A score (AUC) greater than 60.25 indicates intact tactile discriminative sensibility (relative to normative data). Impaired tactile discrimination is suggested if the area under the curve score is less than or equal to 60.25.

**Table 2:** Accuracy of predicting TDT scores from stroke participants using resting-sate functional connectivity. Note: Significant *p* values are in bold font.

Number of Brain Regions	Features	Regression Method	Correlation Coefficient (*r*)	*p* Value
90	LOFC	LR	0.28	**0.038**
		SVR	0.31	**0.024**
	LOFC + HOFC	LR	0.45	**0.002**
		SVR	0.54	**0.0002**

Note: LOFC: low-order functional connectivity; LOFC + HOFC: low-order functional connectivity + high-order functional connectivity; LR: linear regression; SVR: support vector regression.

## Data Availability

Data are available upon reasonable request from the senior author LMC.

## Appendix A

Names and corresponding abbreviations of the brain regions involved in the selected features.

**Abbr.**	**Brain Region**	**Abbr.**	**Brain Region**
CUN	Cuneus	PreCG	Precentral gyrus
FFG	Fusiform gyrus	PoCG	Postcentral gyrus
IFGoperc	Inferior frontal gyrus-opercular	PUT	Putamen
INS	Insula	ROL	Rolandic operculum
IOG	Inferior occipital gyrus	SFGdor	Superior frontal gyrus-dorsal part
IPL	Inferior parietal lobule	SFGmed	Superior frontal gyrus-medial part
ITG	Inferior temporal gyrus	SMG	SupraMarginal gyrus
PAL	Pallidum	TPOmid	Temporal pole-middle
PCUN	Precuneus		
